# Characterization of Influenza Vaccine Hemagglutinin Complexes by Cryo-Electron Microscopy and Image Analyses Reveals Structural Polymorphisms

**DOI:** 10.1128/CVI.00085-16

**Published:** 2016-06-06

**Authors:** Dustin M. McCraw, John R. Gallagher, Audray K. Harris

**Affiliations:** Laboratory of Infectious Diseases, National Institute of Allergy and Infectious Diseases, National Institutes of Health, Bethesda, Maryland, USA; Duke University Medical Center

## Abstract

Influenza virus afflicts millions of people worldwide on an annual basis. There is an ever-present risk that animal viruses will cross the species barrier to cause epidemics and pandemics resulting in great morbidity and mortality. Zoonosis outbreaks, such as the H7N9 outbreak, underscore the need to better understand the molecular organization of viral immunogens, such as recombinant influenza virus hemagglutinin (HA) proteins, used in influenza virus subunit vaccines in order to optimize vaccine efficacy. Here, using cryo-electron microscopy and image analysis, we show that recombinant H7 HA in vaccines formed macromolecular complexes consisting of variable numbers of HA subunits (range, 6 to 8). In addition, HA complexes were distributed across at least four distinct structural classes (polymorphisms). Three-dimensional (3D) reconstruction and molecular modeling indicated that HA was in the prefusion state and suggested that the oligomerization and the structural polymorphisms observed were due to hydrophobic interactions involving the transmembrane regions. These experiments suggest that characterization of the molecular structures of influenza virus HA complexes used in subunit vaccines will lead to better understanding of the differences in vaccine efficacy and to the optimization of subunit vaccines to prevent influenza virus infection.

## INTRODUCTION

The influenza virus is a negative-sense enveloped virus that infects millions of people globally on an annual basis. Annual vaccination is required due to the constant change in the antigenic composition of hemagglutinin (HA), the major antigenic constituent on the viral membrane (1, 2). HAs are classified into 18 antigenic subtypes (H1 to H18) (3, 4). Influenza viruses with subtypes H1, H2, and H3 have caused global epidemics and pandemics in the human population (5). However, other subtypes can infect humans. Influenza has a zoonotic nature, allowing animal viruses, against which the human population has no preexisting immunity, to infect humans. An example of this type of zoonosis was the H7N9 influenza virus that infected humans in 2013, a virus against which seasonal vaccines could not provide protection at that time (6–9).

However, later studies indicated that H7 subtype vaccines were efficacious against the H7N9 virus (10–14). These vaccines consisted of chemically inactivated and noninactivated formulations of viruses and subunit vaccines. Subunit vaccines contain either viral glycoproteins isolated from inactivated viruses (13) or purified influenza virus HA from recombinant protein expression systems (11, 14). HA proteins isolated from influenza virus can form complexes that are referred to as rosettes, protein micelles, or glycoprotein subunits (15, 16). Here we refer to recombinant HA proteins as hemagglutinin complexes (14, 17, 18).

The protomer of trimeric HA is HA0, which is cleaved into disulfide-linked HA1 and HA2 proteins. Three copies of the protomer form the trimeric HA molecule (19, 20). HA1 forms an apical globular region, while extended regions of HA1 and HA2 form the stem region. HA2 also contains a transmembrane region located near the C terminus, which embeds HA in the viral membrane (21). HA mediates viral entry via a low-pH-mediated conformational change that leads to membrane fusion through conversion from a prefusion to a postfusion state (20, 22–24).

Antibodies against HA efficiently neutralize influenza virus. Thus, HA is the principal antigen in influenza vaccines. Recombinant HA vaccines are advantageous because they are not derived from chemical inactivation processes, which can chemically modify viral surface antigens (sAg) (25), and recombinant proteins do not require the time needed to produce the influenza viruses used in the virus-based influenza vaccine formulations (26, 27). While the 3-dimensional (3D) molecular organization of HA and the orientation of HA molecules on the surfaces of viruses have been studied (21, 28), the molecular structure and organization of the recombinant HA complexes used in influenza vaccines have not been studied in great detail. Also, the conformation and mechanism of oligomerization of HA molecules within recombinant HA complexes have not been addressed.

In this study, we used cryo-electron microscopy (cryo-EM) and image analysis to identify and characterize the polymorphisms of H7 HA complexes. HA was not cleaved into HA1 and HA2 but remained as HA0. HA complexes were distributed into four major classes with variable numbers of HA molecules (range, 6 to 8) per complex. The molecules did not present any rigid symmetry but had “starfish-like” motifs. Averaging methods were used to derive information about the orientations of constituent HA molecules within the complexes and to obtain a 3D density map at ∼25 Å resolution. Using the reconstructed density map and molecular modeling with coordinates, we determined that HA was in a prefusion conformation. Models of molecular orientations showed the transmembrane regions emanating from the centers of the complexes. This suggested that nonspecific hydrophobic interactions were the basis for both the polymorphisms and the oligomerization of HA complexes. The ability to characterize the organization of H7 HA molecules within recombinant HA complexes used in vaccine formulations could be extended to other HA subtypes and could be used to investigate the various stabilities and efficacies of subunit vaccines of differing compositions. Furthermore, structural studies of subunit vaccines may describe correlates of immunogenicity for influenza virus HA and other viral glycoproteins used in subunit vaccine formulations.

## MATERIALS AND METHODS

### SDS-PAGE and immunoblotting.

Hemagglutinin proteins were obtained from Protein Sciences Corporation (Meriden, CT) and were recombinant full-length H7 proteins of the A/Netherlands/219/2003 (H7N7) and A/Anhui/1/1013 (H7N9) influenza viruses. The proteins were in phosphate-buffered saline (PBS) and were all produced from a proprietary baculovirus expression system (Protein Sciences Corporation) used to manufacture antigens for commercial influenza vaccines. SDS-PAGE analysis was carried out to assess the purity and HA0 cleavage states of hemagglutinin. For reducing conditions, samples contained the reducing agent dithiothreitol (DTT) at final concentration of 100 mM. For SDS-PAGE under heating conditions, samples were heated at 95°C for 10 min; for nonheating conditions, samples were left at room temperature. Overnight Coomassie blue staining was used to visualize the protein bands of SDS-PAGE gels. For nonreducing or nonheating conditions for the immunoblotting of antigens, no DTT or heating was used during SDS-PAGE before transfer to nitrocellulose membranes. For immunoblotting, the primary antibody was the anti-H7 mouse monoclonal antibody (MAb) InA414 (anti-H7N7) from Novus Biologicals (Littleton, CO). Immunodetection and development were carried out using alkaline phosphatase-conjugated secondary antibodies and the chromogenic substrate NBT (nitroblue tetrazolium)/BCIP (5-bromo-4-chloro-3-indolylphosphate) (Thermo Fisher Scientific, Waltham, MA).

### Cryo-electron microscopy.

For screening by negative staining, samples (4 μl at 100 μg/ml) were applied to continuous carbon grids prepared by glow discharge, stained with 4% uranyl acetate, air-dried at room temperature, and imaged under cryo-conditions like vitrified samples (see below). For cryo-electron microscopy, samples (2 μl at 500 μg/ml in PBS) with no heavy metal stain added were applied to holey carbon films (Quantifoil, Großlöbichau, Germany) and were plunge-frozen using a Vitrobot Mark IV system (FEI Company, Hillsboro, OR). A Titan Krios electron microscope (FEI Company, Hillsboro, OR) operated at liquid nitrogen temperatures and at 300 kV was used to digitally collect images via EPU on a 4,096- by 4,096-pixel charge-coupled-device (CCD) camera (Gatan Inc., Warrendale, PA) at a pixel size of 1.15 Å. The electron doses ranged from ∼10 to 20 e^−^/Å^2^, with defocus values ranging from ∼2.5 to ∼5.5 μm. Cryo-images indicating drift and astigmatism were excluded from further analysis. For further analyses, 5,299 cryo-images were visually screened for the presence of particles.

### Particle boxing and 2D classification.

Because automated methods of particle boxing (picking) failed, as judged by high numbers of false-positive results, manual boxing was carried out to obtain well-separated (i.e., nonoverlapping) particles by screening the cryo-images (*n* = 5,299) and using a box size of 600 Å. Particles (*n* = 3,877) were centered, aligned, and averaged. The 2D circular average was then calculated. To measure the diameter from the 1D profile, the relative intensity along the *x* axis was plotted against the distance from the center (radius) by use of the Bsoft software package (29). 1D profiles for 2D class averages and 3D rotational average profiles were calculated similarly. Complexes were assigned to 2D classes by use of Relion (30). Convergence was considered reached with the assignment of 3,875 of 3,877 particle images (99.9%) to 10 2D classes. The four most populous 2D classes were subjected to 1D profile analysis in order to estimate diameters (Bsoft).

### Computational estimation of numbers of HA molecules in complexes.

In order to estimate the numbers of protrusions (i.e., HA molecules) in the 2D class average images of the most populous classes, libraries of rotated images were created and were then correlated (Bsoft). The number of units was varied to approximate 2, 3, 4, 5, 6, 7, 8, and 9 asymmetric units per 360°, resulting in images rotated by corresponding degree angles (0, 40, 45, 51.42, 60, 72, 80, 90, 102.84, 120, 135, 144, 154.6, 160, 180, 200, 205.68, 216, 225, 240, 250, 257.1, 270, 280, 288, 300, 308.52, 315, 320, and 360°). The correlation maximum from the plots was taken as the number of units (molecules) in the 2D class average image of HA complexes of a particular class.

### 3D reconstruction and molecular modeling.

Because automated methods of particle boxing (picking) failed to pick out the individual protrusions (HA molecules) from the complexes due to overlap, as judged by a high number of false-positive results, manual boxing was used to obtain well-separated HA protrusions for determination of the 3D structural state of HA in the complexes. For the screening of complexes, a box size of 185.12 Å was used to select individual HA protrusions from the complexes. A total of 1,327 individual protrusions (HA molecules) were selected manually and were subjected to further analysis. Particles were subjected first to global shape analysis, via calculation of a 3D average and subsequent profile analysis, and then to 3D reconstruction with the molecular docking of coordinates into the 3D map. 3D structural analyses were carried with Bsoft, EMAN2, and Relion, and molecular docking was carried out with the Chimera software packages (29–32).

## RESULTS

### Hemagglutinins as uncleaved HA0.

**FIG 1:**
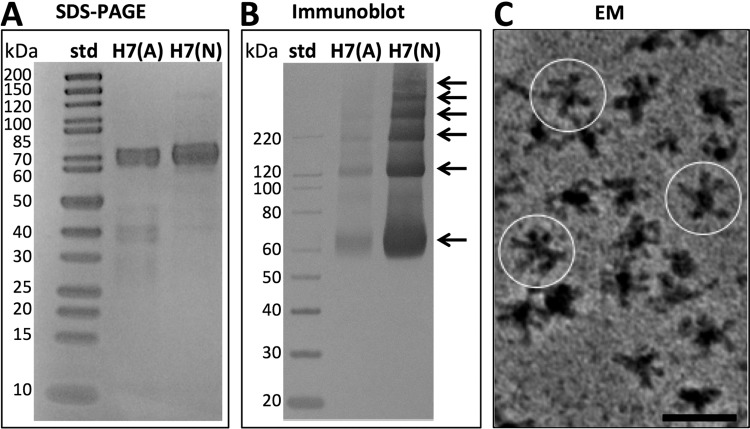
Biochemical characterization of H7 hemagglutinin (HA). (A) SDS-PAGE analysis of purified HA proteins under denaturing and reducing conditions. The H7 proteins from the A/Anhui/01/2013 (H7N9) and A/Netherlands/219/2003 (H7N7) influenza viruses are designated H7(A) and H7(N), respectively. The first lane contains molecular mass standards (std); the second and third lanes contain purified HA proteins. (B) Immunoblot analysis of HA oligomerization under nondenaturing and nonreducing conditions. The first lane contains immunoblot standards; the second and third lanes contain HA proteins. Arrows indicate a ladder of at least six protein bands visible above background. (C) Image of a field of H7 (Netherlands) HA complexes revealed by negative-staining electron microscopy (EM) with a heavy metal stain, uranyl acetate. Image contrast is shown, with black areas representing proteins. Several complexes are circled. Bar, 50 nm.

To determine the cleavage status of the HA1–HA2 cleavage site of the hemagglutinin (HA) proteins, we used SDS-PAGE analysis. This method allowed us to define the relative purity and proportions of HA0, HA1, and HA2 proteins. For both H7 HA proteins, from the Anhui and Netherlands influenza A viruses, protein bands were observed at an apparent molecular mass of about 70 kDa, which is the approximate molecular mass of HA0 (Fig. 1A). Bands for HA1 and HA2 would be expected to appear at about 50 kDa and 20 kDa, respectively. These bands were not observed. The proteins were judged to be >90% and 95% pure by densitometry. Assessment of protein purity and homogeneity was important for further analysis of HA by electron microscopy and immunoblotting, because the purity level ruled out both large amounts of contaminating proteins and heterogeneously cleaved HA proteins, which would hamper further structural analysis and interpretation.

### HA0 protomer detection and HA complexes.

HA proteins have conserved cysteine residues (see Fig. S1 in the supplemental material), and their trimeric structures indicate inter- and intramolecular disulfide bonds within the HA1 and HA2 regions (33, 34). Thus, in order to address the effect of disulfide reduction on HA conformation and oligomerization via immunoblotting, we screened a panel of H7 antibodies under various reducing and nonreducing conditions and identified a monoclonal antibody (InA414) with a conformationally dependent epitope that required nonheating and nonreducing conditions (see Fig. S2 and S3 in the supplemental material). HA0 protomers were detected via immunoblotting using MAb InA414 (Fig. 1B). Six bands were visually apparent in the immunoblot (Fig. 1B). Further analysis of protein banding patterns by densitometry indicated six and four peaks for the H7 Netherlands and H7 Anhui influenza A viruses, respectively (see Fig. S4 in the supplemental material). Based on molecular mass standards ranging from 20 kDa to 220 kDa, the lower three bands of HA had approximate molecular masses of 70 kDa, 120 kDa, and 220 kDa, respectively (Fig. 1B). The top three bands of the six-band H7 Netherlands influenza A virus ladder were larger than the 220-kDa marker and were judged to be higher-molecular-weight oligomers of HA0 (Fig. 1B). Because six HA0 molecules (six protein bands) could represent two HA trimers decomposed into constituent molecules during gel analysis to form a ladder (Fig. 1B), electron microscopy was used to observe HA0 complexes. The H7 Netherlands virus contained isolated molecular complexes, as observed by negative-staining electron microscopy (Fig. 1C). The complexes were not compact or spherical structures but appeared as starfish-like structures with appendages protruding from a central point. Several complexes are circled in Fig. 1C. Similar HA complexes were seen for the H7 Anhui virus by cryo-electron microscopy and were subjected to further 2D and 3D image analyses.

### Molecular organization of HA complexes by cryo-electron microscopy.

**FIG 2:**
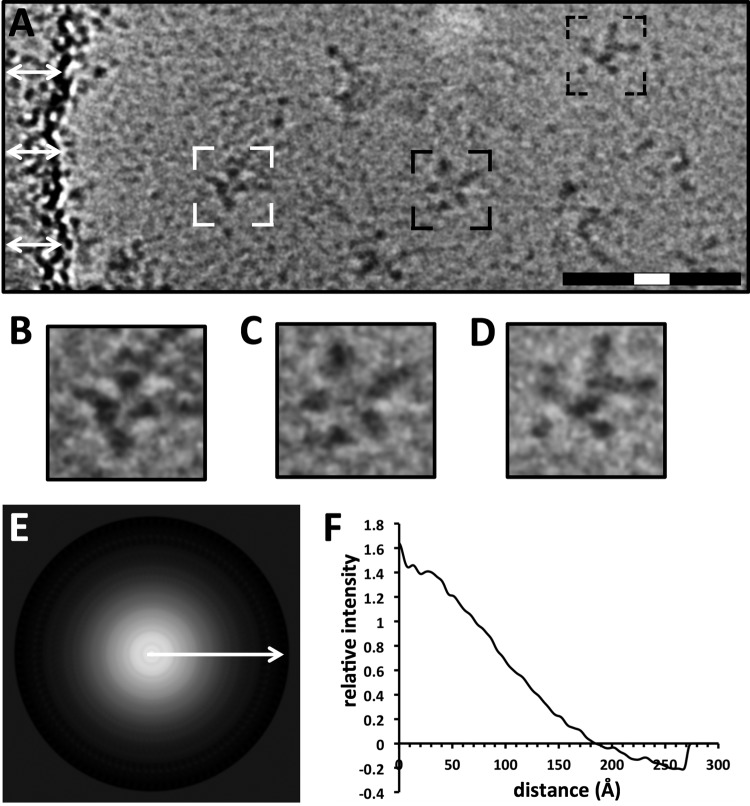
Analysis of Anhui H7 virus HA by cryo-electron microscopy and image analysis. (A) Image of a field of HA complexes of the Anhui H7 virus observed by cryo-electron microscopy in vitreous ice. White arrows indicate the edge of the carbon film substrate. Black areas represent density contrast. The HA complexes shown in panels B to D are outlined. Full bar, 100 nm; white portion of bar, 20 nm. (B to D) Zoom-in views of HA complexes, with dot-shaped and bar-shaped densities representing mostly apical (top view) (B), apical and lateral (side view) (C), and lateral (D) orientations of HA complexes. (E) Circular average of HA complexes from cryo-electron microscopic images. The signal is represented as positive (white). The arrow indicates the direction of the subsequent profile analysis of the radius. (F) One-dimensional radial profile of the circular average, used to estimate the relative diameter of the particle population.

While negative-staining techniques in electron microscopy produce high-contrast images through heavy metal counterstaining, the protein sample is subject to dehydration, and deposition of the negative stain can introduce irregularities into individual particles that antagonize further image analysis. Thus, to understand the molecular organization of HA complexes, we analyzed Anhui H7 virus HA complexes by cryo-electron microscopy and image analysis. HA complexes embedded in vitreous ice appeared as structures with protruding appendages, resembling starfish, by cryo-electron microscopy (Fig. 2A).

Constituent appendages appeared as dark dots or bar shapes (Fig. 2A to D). Observation of the distribution of dot-shaped (HA head domain) and bar-shaped (HA stem domain) densities among the HA complexes suggested the possibility of variable orientations of constituent HA molecules (Fig. 2A to D). In cryo-electron microscopy, a sample is applied to a porous carbon film. Imaging conditions are best when the protein is suspended in vitreous ice within holes of the carbon film (see Fig. S5A in the supplemental material), but some protein complexes adhere directly to the carbon film, where image contrast is reduced (see Fig. S5B). HA complexes were distributed into vitreous ice but had a tendency to bind to the carbon film (see Fig. S5). For image analysis, we excluded complexes that adhered to the carbon film and complexes that overlapped in the 2D projected image. Thus, >5,000 2D cryo-images had to be collected and manually screened in order to obtain ∼4,000 individual, manually boxed images of HA complexes sufficient for analysis of the sizes and molecular organizations of HA complexes (see Materials and Methods). The average size of the HA complex was estimated by a circular average and the derived 1D profile curve (Fig. 2E and F). The 1D profile curve had a smooth falloff to a radius of about 180 Å (Fig. 2F). Thus, the average population diameter (*d* = 2*r*) of the HA complexes was estimated to be about 360 Å (36 nm) (Fig. 2F).

### HA complex classification and analysis.

**FIG 3:**
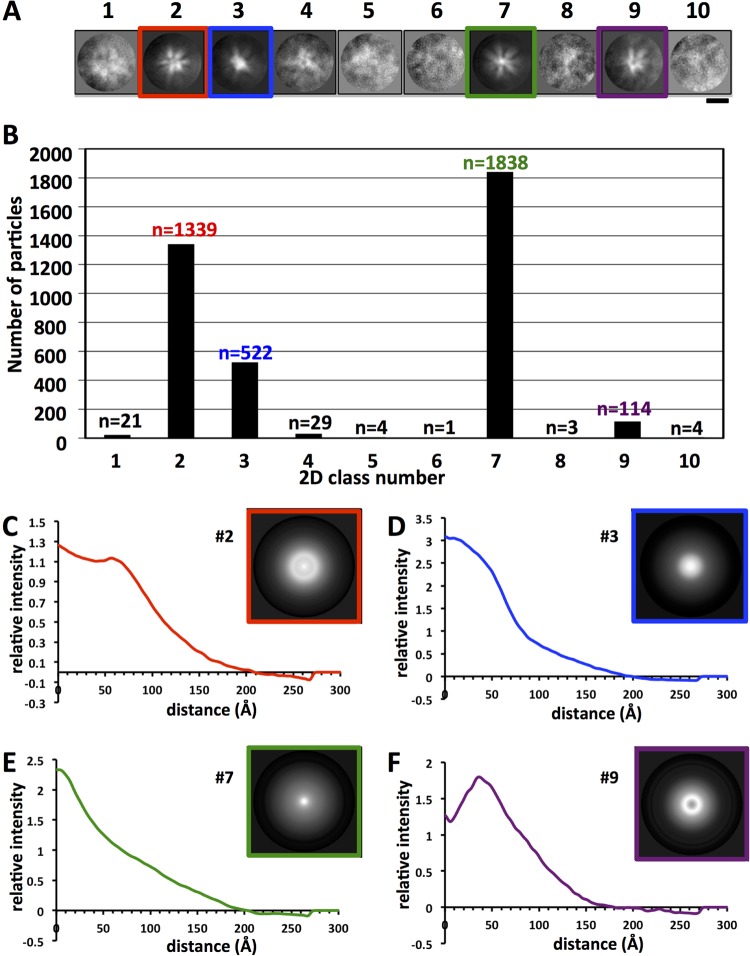
Analyses of HA complexes by 2D image classification and radial profile curves. (A) Gallery of 10 reference-free 2D classes derived from computational analysis of cryo-electron microscopic images of HA complexes (*n* = 3,875). The most populated structural classes are framed in red, blue, green, or purple. Bar, 20 nm. Protein is shown in white. (B) Distribution of 3,875 individual HA complexes assigned by computational analysis to the 10 2D classes. The number of complexes in each class is given above the bar. (C to F) One-dimensional profile curves of the four most populated 2D classes, classes 2, 3, 7, and 9, respectively. The profile curve is the radial density intensity trace from the circular average (inset) of each class, used to measure the relative diameter and density distribution of each class.

Molecular complexes often have rigid symmetry that can be observed by 2D image classification of macromolecules. To answer the questions of whether there is rigid symmetry and whether there are discernibly different subsets of structures, we used computational 2D image classification and analyzed the resulting classes for the sizes and numbers of constituent HA molecules. HA complexes (*n* = 3,875) were distributed into 10 2D classes (classes 1 through 10) (Fig. 3A). Each class had a unique appearance. Six of the classes (classes 1, 4, 5, 6, 8, and 10) were poorly convergent and contained <100 constituent particles (Fig. 3B). The most populated classes were class 7 (*n* = 1,838), class 2 (*n* = 1,339), class 3 (*n* = 522), and class 9 (*n* = 114). Their class average images displayed starfish-like structures, with appendages protruding from the particle center, and with no apparent symmetry, indicating structural polymorphism.

To assess class diameter, 2D circular averages of the four most populous classes were calculated and were used to produce 1D profile curves for measurement of their radii. 1D profile curves appeared distinct in shape. However, two general types of 2D density profile curves calculated from circular averages were observed (Fig. 3C to F). Classes 2 and 9 had observed density peaks at some distance from zero (at approximately 55 and 35 Å, respectively) (Fig. 3C and F), while classes 3 and 7 had density peaks near the center, set to zero distance (Fig. 3D and E). The radii of the classes differed and were estimated as ∼ 41.1 nm, 38.6 nm, 39.7 nm, and 35.1 nm for classes 2, 3, 7, and 9, respectively (Fig. 3C to F). Based on the purity of the sample, antibody reactivity, and size, the constituent protrusions/appendages within the complexes were assigned to HA molecules.

### Estimation of the number of constituent HA molecules per class.

**FIG 4:**
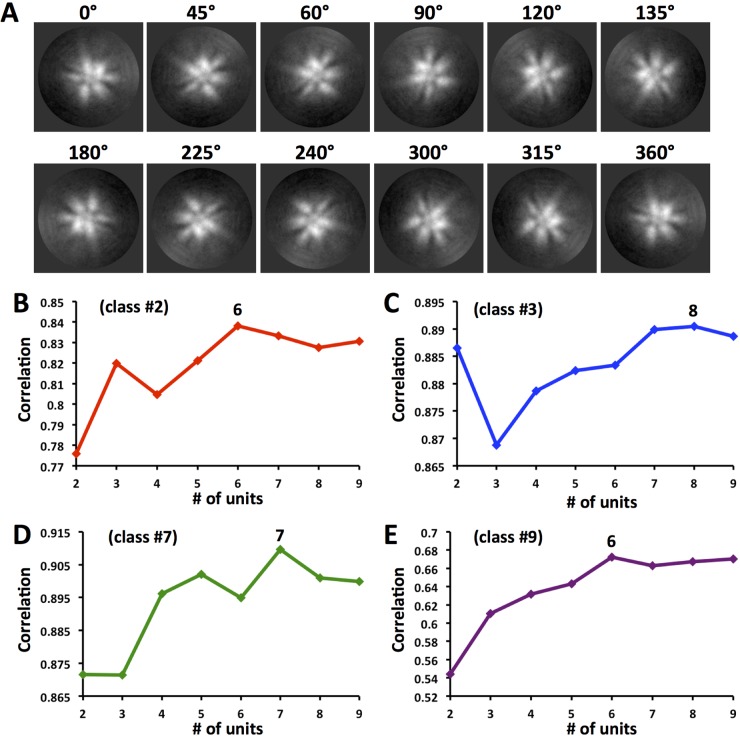
Estimation of the number of protruding appendages (units) per 2D class of HA complexes by image rotation and correlation. (A) Montage of some representative images from the rotation image library for class 2 of the HA complex. Images are rotated by degree increments as shown above each panel. Protein is represented as white. (B to E) Average correlation curves of HA complexes of 2D classes 2, 3, 7, and 9, shown in red, blue, green, and purple, respectively. Rotated images calculated to have increasing numbers of units (asymmetric units per 360°) are correlated. The maximum correlation peak denotes the optimal number of protruding appendages (units) for that 2D class of HA complex. The correlation maximum (6, 8, 7, or 6 units) is given above the analysis curve for each class.

Classes displayed differences in the number of HA molecules emanating from the complex (Fig. 3A). Because 2D image classification produced classes with discernible HA density protrusions even in the absence of rigid symmetry, further computational analyses were done to estimate the number of protruding HA densities within these classes (Fig. 3A). We developed a strategy using image rotation and correlation to estimate the number of HA protrusions (assumed as slightly shifted asymmetric units) per 360° in 2D classes 2, 3, 7, and 9. For each class, an image library was created by rotating each image at angular increments within the rotation range of 0 to 360° (see Materials and Methods). Image rotation resulted in the rotation of protrusions around the center. As expected, 0° and 360° images were identical, as indicated by some examples of rotated images for class 2 (Fig. 4A). Classes had different estimated numbers of HA protrusions based on the highest correlation peak from the correlation plots (Fig. 4B to E). The number of protrusions (units) ranged from 6 to 8 per 360° rotation. Classes 2 and 9 were estimated to have 6 units, while class 7 had 7 units and class 3 had the highest estimated number, 8 units (Fig. 4B to E).

### Molecular conformation of HA molecules within HA complexes.

**FIG 5:**
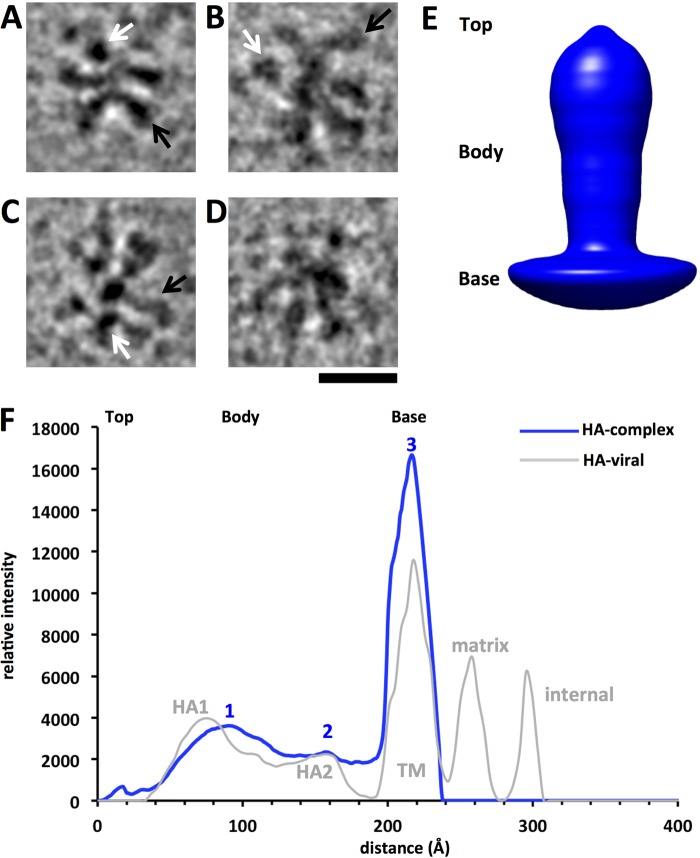
Molecular orientations and conformation of HA within complexes by cryo-electron microscopy and profile analysis. (A to D) Gallery of individual HA complexes consisting of individual HA molecules computationally filtered to improve contrast. Black arrows indicate bar-shaped densities, representing approximately lateral views of constituent HA molecules, and white arrows indicate dot-shaped densities, representing approximately apical views of HA molecules, within the complexes. Protein is depicted in black. Bar, 20 nm. (E) 3D rotational average structure of constituent HA molecules picked from complexes, shown as a solid surface rendering. Regions are designated as the top, body, and base. (F) One-dimensional profile curve of the average structure shown in panel E, allowing the assessment of structural features and size. The three major peaks are labeled 1, 2, and 3, respectively. For comparison, a 1D profile derived from a viral HA 3D map (28) is shown with structural regions labeled. TM, transmembrane region.

In order to address the questions of HA orientation and the 3D molecular conformation of the HA molecules within HA complexes, we used 3D reconstruction techniques to calculate a circular 3D average and then a refined 3D reconstruction map. Computational filtering of images to improve the visual contrast of HA complexes was used to help observe the relative orientations of HA molecules within the complexes (Fig. 5A to D). Complexes had constituent HA molecules that appeared to be in different apical, lateral, and tilted arrangements with respect to the image plane. In some instances, lateral projections of the HA molecules within the complexes resolved as peanut-shaped densities (Fig. 5A to C, black arrows). In apical projections, the HA molecules within the HA complexes appeared as dark dots or triangle-like shapes (Fig. 5A to C, white arrows).

In cryo-electron microscopy, samples are quickly frozen and are suspended in a 3D layer of vitreous ice (Fig. 2A). Cryo-EM images are 2D projection images resulting from the electron beam transmitting through the 3D structure of the sample in a frozen hydrated state (see Fig. S6 in the supplemental material). Schematics of apical and lateral projection images from HA molecules are shown in Fig. S6 in the supplemental material. Although the raw images (Fig. 2A) and 2D classes (Fig. 3A) might give the impression of planar HA complexes, notable details indicated that the complexes were 3-dimensional. Views of the HA molecules in apparent apical (Fig. 5, white arrows) and lateral (black arrows) orientations were observed, in agreement with a nonplanar configuration of the HA complexes (Fig. 5A to D). The radial density observed in the 2D class average images also indicated that the complexes were 3-dimensional. The highest density in 2D class average images was at or near the center of the complex, a pattern consistent with a projection image of a globular structure (Fig. 3C to F). Thus, the HA molecules are likely to be irregularly distributed around the center of the complex in all 3 dimensions.

Individual HA appendages from the HA complexes were boxed, and a 3D circular average was calculated. The 3D average molecule had a body with a bilobed shape on top of a base (Fig. 5E). The 3D average structure consisted of three molecular regions, as detected by the shape of its density profile curve (Fig. 5F, blue curve). The profile curve derived from the 3D average structure was similar to the profile curve derived from a viral HA map that had been solved by cryo-electron microscopy previously (28) (Fig. 5F, gray curve). Peaks 1, 2, and 3 of the HA complex data corresponded approximately to the peaks for the HA1, HA2, and transmembrane regions of viral HA, respectively (Fig. 5F). The lengths of the ectodomain and the transmembrane region of the average HA molecule were measured from the widths of peaks 1, 2, and 3. The ectodomain assigned to peaks 1 and 2 spanned about 158 Å (15.8 nm), and the transmembrane region assigned to peak 3 had a width of about 40 Å (4 nm) (Fig. 5F, blue curve).

**FIG 6:**
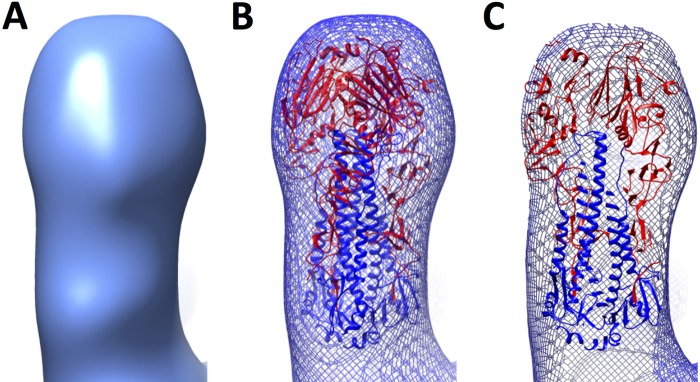
3D reconstruction (3D map) derived from individual HA molecules boxed out from the complexes. (A) The 3D map is shown as a solid surface rendering. (B) The 3D map is shown as a wire mesh with docked coordinates of the H7 virus HA ectodomain from the Protein Data Bank. HA1 is shown in red, while HA2 is depicted as blue ribbons. The transmembrane domain is not shown, because no coordinates are available. (C) Similar to panel B, but with a sliced view.

**FIG 7:**
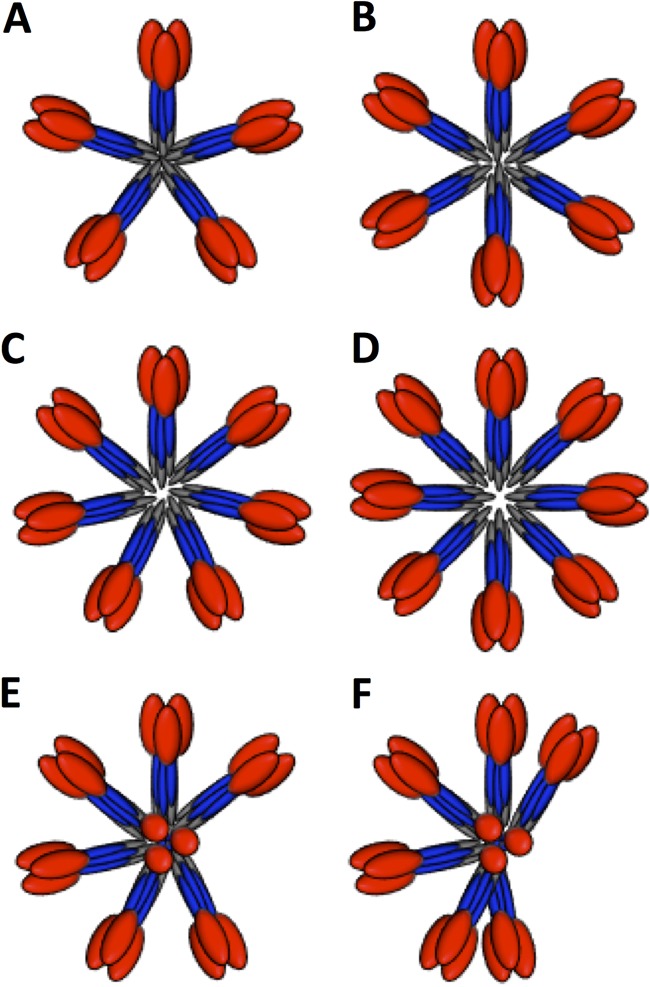
Schematic models of HA complexes to illustrate the positional variations of constituent HA molecules. (A to D) Schematics of five, six, seven, and eight HA trimers, respectively, in planar starfish-like arrangements. (E) Schematic of seven HA trimers in an asymmetric starfish-like arrangement with one molecule in an upward-facing position. (F) Schematic of seven HA trimers in an asymmetric starfish-like arrangement with one molecule in an upward-facing position and various distances between lateral molecules. In panels E and F, one HA molecule is perpendicular in order to indicate a top axial view as opposed to lateral views. In all the schematic models, HA1 is shown in red, HA2 in blue, and transmembrane regions in gray.

To understand the molecular disposition of the HA1 and HA2 regions in HA complexes, a 3D reconstruction was determined from computationally boxed appendages of the HA complexes. The refined 3D reconstruction was bilobed (Fig. 6A). The lobe at the top was larger than the lower lobe, which gave a peanut-shaped appearance to the 3D map (Fig. 6A). The docking of H7 virus HA coordinates onto the 3D map matched the top, larger lobe to the HA1 head region (red), and the lower lobe to the HA2 stem region (blue), of the coordinates (Fig. 6B). The docking positioned the HA2 C terminus of the ectodomain at the bottom of the 3D map (Fig. 6B and C). This HA orientation was integrated into schematic models of HA complexes with bilobed molecules and transmembrane regions that are proximal to one another and centrally located within the HA complexes (Fig. 7).

## DISCUSSION

In order to answer questions about the molecular organization and conformation of hemagglutinin (HA) complexes used in recombinant subunit influenza vaccines, we studied HA H7 complexes biochemically and by cryo-electron microcopy followed by image analyses and 3D reconstruction. Our results suggested that HA complexes have heterogeneous numbers and arrangements of constituent HA molecules. The 3D map derived from cryo-electron microscopy data displayed a structure with the HA in a prefusion state, with the HA1 globular head oriented at the top of the complex and the HA2 stem region below it, suggesting that the orientation of the transmembrane region is approximately toward the center of the HA complex.

### Hierarchy of molecular interactions within HA complexes.

Because partial cleavage of HA0 during the purification and storage of recombinant HA complexes may reduce vaccine potency (17, 35), we analyzed the banding patterns of the H7 HA proteins by SDS-PAGE and immunoblotting to determine both HA cleavage status and the relative strength of interactions between HA proteins. Single major bands at ∼70 kDa suggested that HAs were homogeneous proteins in the uncleaved HA0 state (Fig. 1A). This is important because proteolytic degradation can decrease antigen stability in HA complex-based vaccines (36). Based on protein sequences, the H7 Anhui and H7 Netherlands influenza A viruses have predicted molecular masses of 62.1 kDa and 62.5 kDa, respectively, and the higher apparent molecular masses of HA are likely explained by glycosylation. HA molecules formed oligomers, as indicated by ladders of bands in immunoblots. HA complexes appeared as starfish-like clusters of HA molecules in cryo-electron microscopic images (Fig. 2A and 3A). We estimated by cryo-electron microscopy image processing that an HA complex had as many as 8 HA molecules (Fig. 4C). This would represent 24 (8 × 3) protomers of HA. However, by SDS-PAGE and immunoblotting, only 6 bands were observed, and these can be interpreted as representing 1, 2, 3, 4, 5, and 6 protomers of HA0 (Fig. 1B). This implies that the SDS conditions affected the complex but that not all interactions within the complex were abrogated to a single HA monomer state, as judged by the appearance of bands representing integral weights of HA (Fig. 1B). Detergent is used and removed during the purification of HA0 from the membranes of recombinant systems. This suggests a model for a hierarchy of interactions in which HA complexes may be formed by the association of one HA trimer with another as detergent is removed during the protein purification process.

Redox conditions could also affect the hierarchy of these interactions, because when disulfide bonds were reduced in the presence or absence of heat denaturation conditions, HA oligomers were no longer observed (see Fig. S2 and S3 in the supplemental material). This implies that the integrity of the conserved cysteines forming intra- and intermolecular disulfide bonds of HA is required not only in order to maintain the 3D structure of HA (37) but also to enforce other interactions within the HA complex. Indeed, the formation of nonnative disulfides can reduce the potency of recombinant HA vaccines (35), and certain chemical additives can inhibit the action of nonnative disulfides (38). The correct disposition of disulfide bonds has be studied in other systems, such as hepatitis B surface antigen particles, where cysteine residues are important for assembly and epitope display (39). Our results with MAb InA414 indicate that antibodies with conformational, disulfide-dependent epitopes can be used to assess the proper folding and oligomerization hierarchy of HA molecules within HA complexes.

### Arrangements and numbers of constituent HA molecules within complexes.

Some viral vaccines are composed of recombinant viral antigens arranged as rigid particles, such as the virus-like particles of human papillomavirus (HPV) (40, 41) and sAg particles of hepatitis B virus (42–44), and can have hundreds of copies of the viral antigen. However, the rigidity, flexibility, and number of HA molecules within HA complexes used in influenza vaccines had not been analyzed in detail (17). From 2D classification we would expect to detect rigid symmetrical arrangements of molecules, because such arrangements can be detected by image processing, as shown for symmetrical particles such as those of HPV and designed influenza virus nanoparticles (41, 45). Our results using cryo-electron microscopy and 2D classification indicated nonrigid structures with no apparent consistent symmetry for a population of several thousand HA complexes (Fig. 3A and B). However, HA complexes did fall into different classes, and curve analyses revealed differences between the relative diameters of HA complexes in these classes (Fig. 3). The differences between the shapes and peak maximums of class profile curves suggest that HA complexes can exist in at least two major states: open and closed. In open states, the constituent HA molecules of the complexes are spread out radially from the center like the arms of an open starfish. In closed states, the HA molecules have a more side-by-side or collimated arrangement, like the arms of a closed starfish. Further evidence for this is seen in images of individual complexes, where apparent apical (top view) and lateral (side view) structures of HA molecules emanate from a common center (Fig. 2A to D and 5A to D). For example, particles with more top views of HA could represent a closed state of the HA complex (Fig. 2B), while particles with more lateral views of HA could represent an open state (Fig. 2D). HA complexes with both apical and lateral views of HA molecules could represent a possible third, mixed state (Fig. 2C). Although HA complexes appeared nonrigid, with asymmetric arrangements of constituent HA molecules, they did display HA molecules as protrusions emanating from a common center to give rise to starfish-like arrangements of HA molecules within 2D classes. However, visual counting of the number of HA molecules in images of individual HA complexes (Fig. 2A to D) and 2D classes (Fig. 3A) would be subjective.

Thus, we devised a more objective computational strategy to estimate the number of HA molecules per complex within the 2D classes by correlating rotated images and plotting the correlations (Fig. 4). Also, the number of HA molecules provides information on multivalency via the number of epitopes in the HA complex. This is important because multivalency is thought to improve the immunogenicity of viral antigens (40, 45). The number of estimated HA molecules ranged from 6 to 8 for HA complexes (Fig. 4B to E). Thus, trimeric HA within HA complexes could have a predicted maximum of 24 (3 × 8) epitopes per complex. This number of epitopes is much less than that of influenza virus particles, which have hundreds of HA molecules and epitopes (21, 28). Also, this predicted maximum of 24 epitopes is much less than that for the virus-like particle of HPV vaccine, which has 360 copies of the major capsid protein L1 (40). However, HA complexes can be formulated into efficacious influenza vaccines (17, 18, 46). Moreover, designed octahedral nanoparticles that display epitopes for hemagglutinin can elicit protective antibodies in animal models (45, 47). These octahedral nanoparticles contain 24 copies of the HA polypeptide, like the maximum 24 epitope copies for the HA complex with 8 trimeric HA molecules. Taken together, these results suggest that HA epitopes can be displayed on particles that differ in both the number and the arrangement of constituent HA molecules. However, HA on the influenza virus surface and designed HA nanoparticles were shown to be in the prefusion state by cryo-electron microscopy (28, 45), and in this study, we determined that HA within HA complexes is in the prefusion state, as well.

### HA conformation in a prefusion state.

Because influenza virus HA can exist in prefusion and postfusion states (20, 22, 28), the conformational state of HA within HA complexes was determined by image analysis and 3D reconstruction coupled with molecular modeling. Although structures of prefusion HA have been observed in soluble HA ectodomains without transmembrane regions and in HA with transmembrane regions on viral surfaces (20, 22, 28), the 3D conformation of HA within recombinant HA complexes used as vaccine immunogens had not been addressed in great detail. Previous studies have used heavy metal stains and dehydrated samples fixed to a carbon support, conditions under which structural flattening and deformations can occur. Also, images of only a small number of particles (<20) were studied (11, 36). Our analysis of thousands of images by cryo-electron microscopy of H7 HA complexes showed complexes with constituent protruding densities (Fig. 2A to D and 3A). It is difficult to differentiate between pre- and postfusion states of viral glycoproteins without 3D image analysis (48, 49). Therefore, although the protruding densities suggested an HA shape (Fig. 2A to D and Fig. 3A), we performed 3D reconstruction analyses. These 3D analyses revealed that HA within HA complexes is in a prefusion state conformation (Fig. 5 and 6).

HA undergoes a low-pH-induced conformational change that mediates viral fusion, resulting in a prefusion-to-postfusion structural transition (20, 22, 24). Some broadly neutralizing antibodies target the prefusion state and can inhibit the prefusion-to-postfusion conformational change (50–53). Thus, it is important to assess the pre- or postfusion states of HA within vaccine platforms (11, 17, 46) that use HA complexes as antigens, because neutralizing epitopes can be disrupted or obscured in the postfusion state. In this study, the H7 HA in HA complexes was in the prefusion state (Fig. 6). In contrast, glycoprotein complexes of the F protein (fusion protein) of respiratory syncytial virus in a postfusion state have been used in vaccine preparations (54). This is because the F protein is prone to transition from its prefusion to its postfusion state. However, some neutralizing antibodies are specific for epitopes only in the prefusion state of the F protein (55, 56). Thus, it is possible that different influenza virus HAs may show variable prefusion and postfusion propensities when formulated into HA complexes under different expression and manufacturing conditions. The technical advances reported here in the study of nonrigid and asymmetric HA complexes by 2D and 3D image analyses should prove helpful in further understanding prefusion and postfusion conformations in various glycoprotein complexes used as subunit vaccines.

### Models of HA complexes from distance constraints.

The 3D reconstruction and subsequent molecular docking of the H7 ectodomain coordinates oriented the HA2 C termini, which lead into the transmembrane regions (see Fig. S1 in the supplemental material) toward the centers of the HA complexes (Fig. 6). This result suggests that the HA transmembrane regions within HA complexes are proximal to one another (Fig. 7). These transmembrane domains are absent in the HA ectodomain crystal structures that have been solved (20, 51). However, comparison of structural data from HA complexes with HA on the viral surface suggested the matching of 4-nm transmembrane regions (Fig. 5F), because lipid bilayers span about 4 nm. Comparison of viral HA and HA complex sizes suggests that the transmembrane regions of constituent HA molecules are in close proximity and could engage in hydrophobic interactions (Fig. 7). The relative height of the HA molecule within the complex was about 16 nm, with a 4-nm transmembrane region (Fig. 5F, blue curve). Thus, the estimated total length of a constituent HA molecule within the HA complex would be 20 nm. However, the observed average diameter of the HA complex is 36 nm (Fig. 2F). Thus, if the transmembrane regions were not overlapping but tail to tail, the calculated diameter would be 40 nm. One interpretation of this difference of 4 nm between the calculated (40-nm) and observed (36-nm) diameters is that the transmembrane regions of HA molecules overlap in space within the HA complexes. Accordingly, this distance constraint would help satisfy hydrophobic interactions and would give the observed average diameter (36 nm) for the HA complex (i.e., 2 ectodomains plus 1 overlapping transmembrane region [16 + 16 + 4 = 36 nm]). The overlapping interaction space of the hydrophobic transmembrane regions would explain the polymorphism, because hydrophobic interactions, which are dominated by nonspecific van der Waals interactions, could allow the movement of constituent HA molecules and variable HA numbers within HA complexes (Fig. 7).

No redundant trimer-trimer interactions between the globular head regions of constituent HA molecules within the HA complexes were observed. This is different from the behavior of other symmetrical immunogens, such as protein nanoparticles, which have large areas of buried surfaces between protomers (41, 45). However, this suggests that the ability of different subtypes of HA proteins to form HA complexes may be based on hydrophobic interactions involving the transmembrane regions (18). Interestingly, some sequence variations of HA transmembrane regions may confer additional stability on HA complexes, because some chimeric HA proteins based on transmembrane region exchanges showed enhanced heterosubtypic protection in mice (57).

Characterization of the sizes and organizations of vaccine antigens is important not only for maintaining consistent quality control of vaccine preparations but also for understanding how variations in size and antigen organization may affect vaccine efficacy. There are a number of biophysical methods for characterizing recombinant protein and particulate vaccines, such as X-ray crystallography, small-angle X-ray scattering (SAXS), dynamic light scattering (DLS), and analytical ultracentrifugation. The use of X-ray techniques has been focused on soluble ectodomains of influenza virus HA molecules (20, 58). DLS and analytical ultracentrifugation can provide estimates of the general sizes of particles (36, 59). However, unlike these methods, cryo-electron microscopy allows direct structural imaging of macromolecules in the aqueous phase. In this work, we focused on defining the structural organization of HA complexes by cryo-electron microscopy followed by 2D and 3D image analyses along with molecular modeling. We created methods to determine the number of constituent HA molecules and the conformational state of HA. These cryo-EM methods of HA complex analysis can be integrated into current vaccine development workflows, which make use of biophysical methods to characterize vaccine antigens.

In conclusion, our results from this study of HA complexes suggest that the underlying mechanism of HA oligomerization is hydrophobic interactions involving transmembrane domains and that these hydrophobic interactions could be the basis for the observed structural polymorphism of HA complexes. Structural polymorphism was indicated by variability in both the number and the arrangement of HA molecules within HA complexes. HA was in a prefusion state, suggesting that HA folding and assembly into HA complexes did not trigger the prefusion-to-postfusion transition. The analyses presented here for HA complexes should prove useful in assessing the molecular organization of constituent HA molecules across various influenza virus HA subtypes used in influenza virus subunit vaccines.

## Supplementary Material

Supplemental material
