# Supplementary material for: Characterization of Influenza Vaccine Hemagglutinin Complexes by Cryo-Electron Microscopy and Image Analyses Reveals Structural Polymorphisms
Source: Clin Vaccine Immunol. 2016 Jun 6;23(6):483–95. doi: 10.1128/CVI.00085-16 (PMC4895014; doi:10.1128/CVI.00085-16)
Supplement: Supplemental material [file supp_23_6_483__index.html]

Supplemental material 

# Characterization of Influenza Vaccine Hemagglutinin Complexes by Cryo-Electron Microscopy and Image Analyses Reveals Structural Polymorphisms

## Supplemental material

- Supplemental file 1 -

  Fig. S1. H7 sequence comparison. Fig. S2. Conformational dependence of mAb IN414 detection of H7 hemagglutinin probed by Western blotting. Fig. S3. Disulfide dependence of mAb IN414 detection of H7 hemagglutinin. Fig. S4. Analysis of relative band number and distances of HA ladder by profile analysis of immunoblot. Fig. S5. Examples of the distribution of H7 complexes by cryo-electron microscopy. Fig. S6. Schematic of holey carbon film and particles in vitreous ice by cryo-electron microscopy. Fig. S7. 3D reconstruction of HA compared with prefusion and postfusion HA ectodomain coordinate structures.

  PDF, 745K
