# Supplementary material for: Characterization of Influenza Vaccine Hemagglutinin Complexes by Cryo-Electron Microscopy and Image Analyses Reveals Structural Polymorphisms
Source: Clin Vaccine Immunol. 2016 Jun 6;23(6):483–95. doi: 10.1128/CVI.00085-16 (PMC4895014; doi:10.1128/CVI.00085-16)
Supplement: Supplemental material [file CVI.00085-16_zcd999095358so1.pdf]

```
Netherlands 1    MNTQILVFALVASIPTNADKICLGHHAVSNGTKVNTLTERGVEVVNATETVERTNVPRIC  60
                 MNTQILVFAL+A IPTNADKICLGHHAVSNGTKVNTLTERGVEVVNATETVERTN+PRIC
Anhui       1    MNTQILVFALIAIIPTNADKICLGHHAVSNGTKVNTLTERGVEVVNATETVERTNIPRIC  60

Netherlands 61   SKGKRTVDLGQCGLLGTITGPPQCDQFLEFSADLIIERREGSDVCYPGKFVNEEALRQIL  120
                 SKGKRTVDLGQCGLLGTITGPPQCDQFLEFSADLIIERREGSDVCYPGKFVNEEALRQIL
Anhui       61   SKGKRTVDLGQCGLLGTITGPPQCDQFLEFSADLIIERREGSDVCYPGKFVNEEALRQIL  120

Netherlands 121  RESGGIDKETMGFTYSGIRTNGTTSACRRSGSSFYAEMKWLLSNTDNAAFPQMTKSYKNT  180
                 RESGGIDKE MGFTYSGIRTNG TSACRRSGSSFYAEMKWLLSNTDNAAFPQMTKSYKNT
Anhui       121  RESGGIDKEAMGFTYSGIRTNGATSACRRSGSSFYAEMKWLLSNTDNAAFPQMTKSYKNT  180

Netherlands 181  RKDPALIIWGIHHSGSTTEQTKLYGSGNKLITVGSSNYQQSFVPSPGARPQVNGQSGRID  240
                 RK PALI+WGIHHS ST EQTKLYGSGNKL+TVGSSNYQQSFVPSPGARPQVNG SGRID
Anhui       181  RKSPALIVWGIHHSVSTAEQTKLYGSGNKLVTVGSSNYQQSFVPSPGARPQVNGLSGRID  240

Netherlands 241  FHWLILNPNDTVTFSFNGAFIAPDRASFLRGKSMGIQSEVQVDANCEGDCYHSGGTIISN  300
                 FHWL+LNPNDTVTFSFNGAFIAPDRASFLRGKSMGIQS VQVDANCEGDCYHSGGTIISN
Anhui       241  FHWLMLNPNDTVTFSFNGAFIAPDRASFLRGKSMGIQSGVQVDANCEGDCYHSGGTIISN  300

Netherlands 301  LPFQNINSRAVGKCPRYVKQESLLLATGMKNVPEIPKRRRRGLFGAIAGFIENGWEGLID  360
                 LPFQNI+SRAVGKCPRYVKQ SLLLATGMKNVPEIPK   RGLFGAIAGFIENGWEGLID
Anhui       301  LPFQNIDSRAVGKCPRYVKQRSLLLATGMKNVPEIPK--GRGLFGAIAGFIENGWEGLID  358

Netherlands 361  GNYGFRHQNAQGEGTAADYKSTQSAIDQITGKLNRLIEKTNQQFELIDNEFTEVERQIGN  420
                 GNYGFRHQNAQGEGTAADYKSTQSAIDQITGKLNRLIEKTNQQFELIDNEF EVE+QIGN
Anhui       359  GNYGFRHQNAQGEGTAADYKSTQSAIDQITGKLNRLIEKTNQQFELIDNEFNEVEKQIGN  418

Netherlands 421  VINWTRDSMTEVWSYNAELLVAMENQHTIDLADSEMNKLYERVKRQLRENAEEDGTGCFE  480
                 VINWTRDS+TEVWSYNAELLVAMENQHTIDLADSEM+KLYERVKRQLRENAEEDGTGCFE
Anhui       419  VINWTRDSITEVWSYNAELLVAMENQHTIDLADSEMDKLYERVKRQLRENAEEDGTGCFE  478

Netherlands 481  IFHKCDDDCMASIRNNTYDHSKYREEAIQNRIQIDPVKLSSGYKDVILWFSFGASCFILL  540
                 IFHKCDDDCMASIRNNTYDHSKYREEA+QNRIQIDPVKLSSGYKDVILWFSFGASCFILL
Anhui       479  IFHKCDDDCMASIRNNTYDHSKYREEAMQNRIQIDPVKLSSGYKDVILWFSFGASCFILL  538

Netherlands 541  AIAMGLVFICVKNGNMRCTICI  569
                 AI MGLVFICVKNGNMRCTICI
Anhui       539  AIVMGLVFICVKNGNMRCTICI  560
```

Figure S1

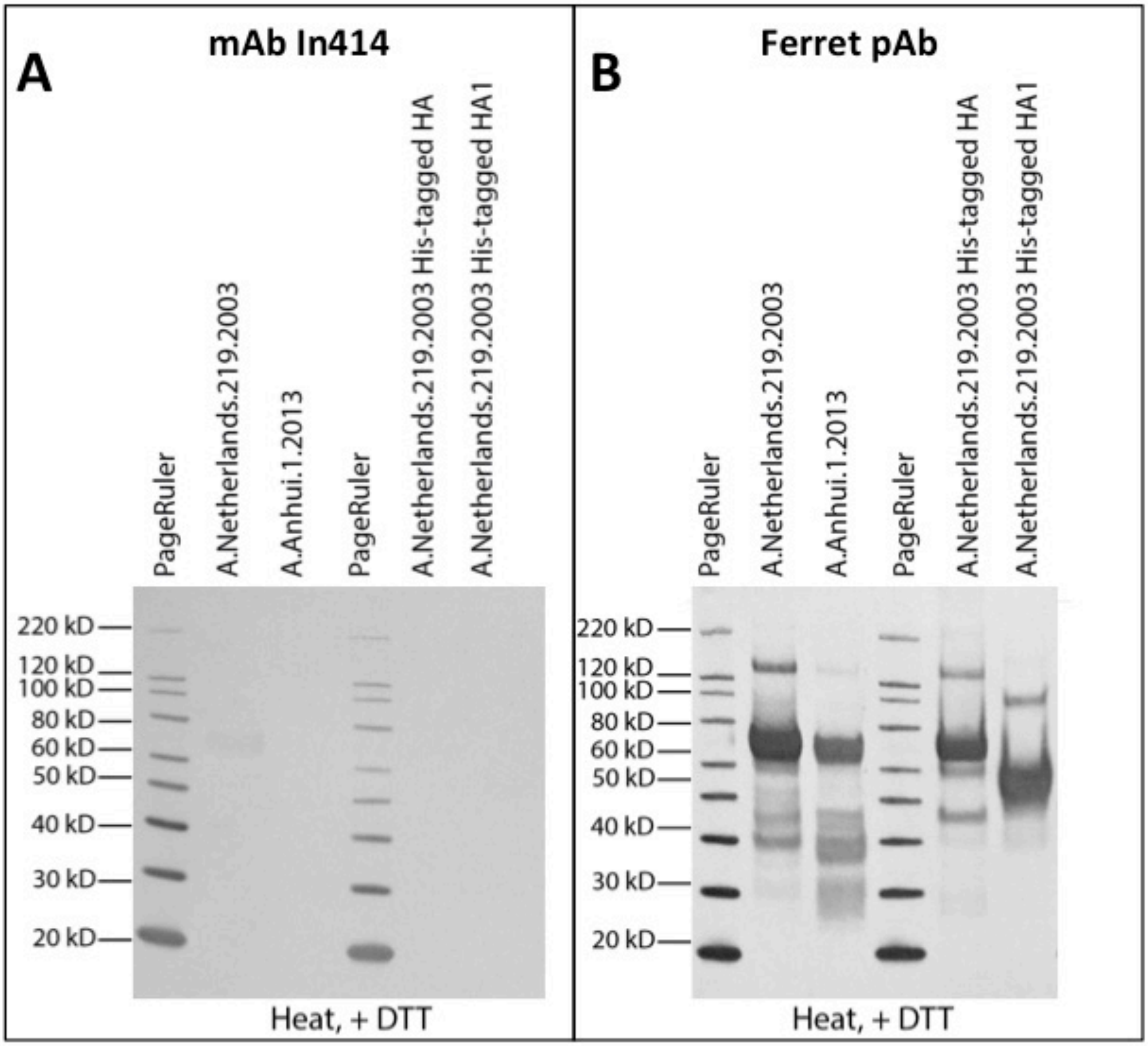
A
mAb In414
PageRuler
A.Netherlands.219.2003
A.Anhui.1.2013
PageRuler
A.Netherlands.219.2003 His-tagged HA
A.Netherlands.219.2003 His-tagged HA1
220 kD
120 kD
100 kD
80 kD
60 kD
50 kD
40 kD
30 kD
20 kD
Heat, + DTT
B
Ferret pAb
PageRuler
A.Netherlands.219.2003
A.Anhui.1.2013
PageRuler
A.Netherlands.219.2003 His-tagged HA
A.Netherlands.219.2003 His-tagged HA1
220 kD
120 kD
100 kD
80 kD
60 kD
50 kD
40 kD
30 kD
20 kD
Heat, + DTT

Figure S2

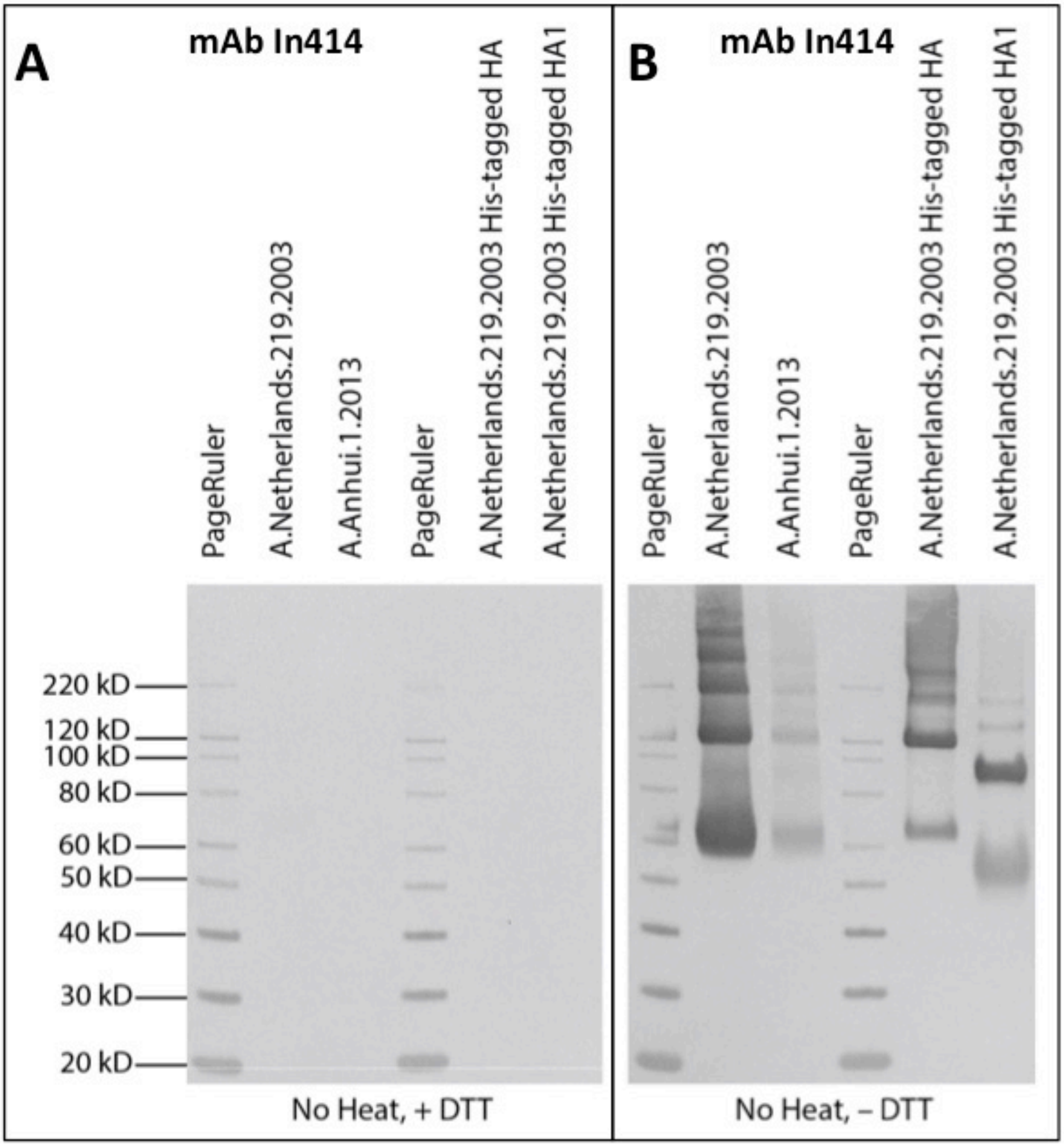
A
mAb In414
PageRuler
A.Netherlands.219.2003
A.Anhui.1.2013
PageRuler
A.Netherlands.219.2003 His-tagged HA
A.Netherlands.219.2003 His-tagged HA1
220 kD
120 kD
100 kD
80 kD
60 kD
50 kD
40 kD
30 kD
20 kD
No Heat, + DTT
B
mAb In414
PageRuler
A.Netherlands.219.2003
A.Anhui.1.2013
PageRuler
A.Netherlands.219.2003 His-tagged HA
A.Netherlands.219.2003 His-tagged HA1
No Heat, – DTT

Figure S3

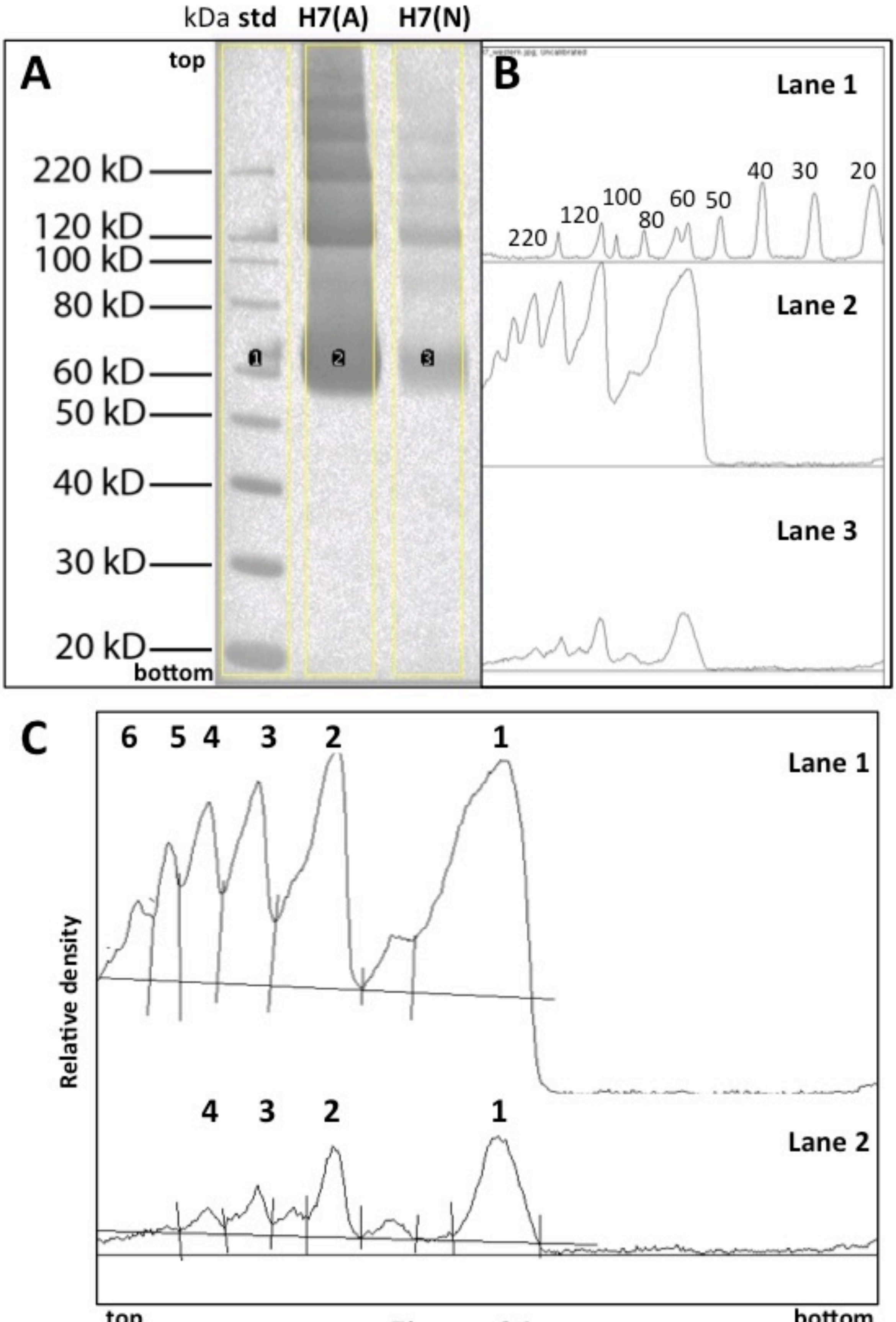
kDa std H7(A) H7(N)
A
top
220 kD
120 kD
100 kD
80 kD
60 kD
50 kD
40 kD
30 kD
20 kD
bottom
1
2
3
B
Lane 1
220
120
100
80
60
50
40
30
20
Lane 2
Lane 3
C
6 5 4 3 2 1
Lane 1
Relative density
4 3 2 1
Lane 2
top
bottom

Figure S4

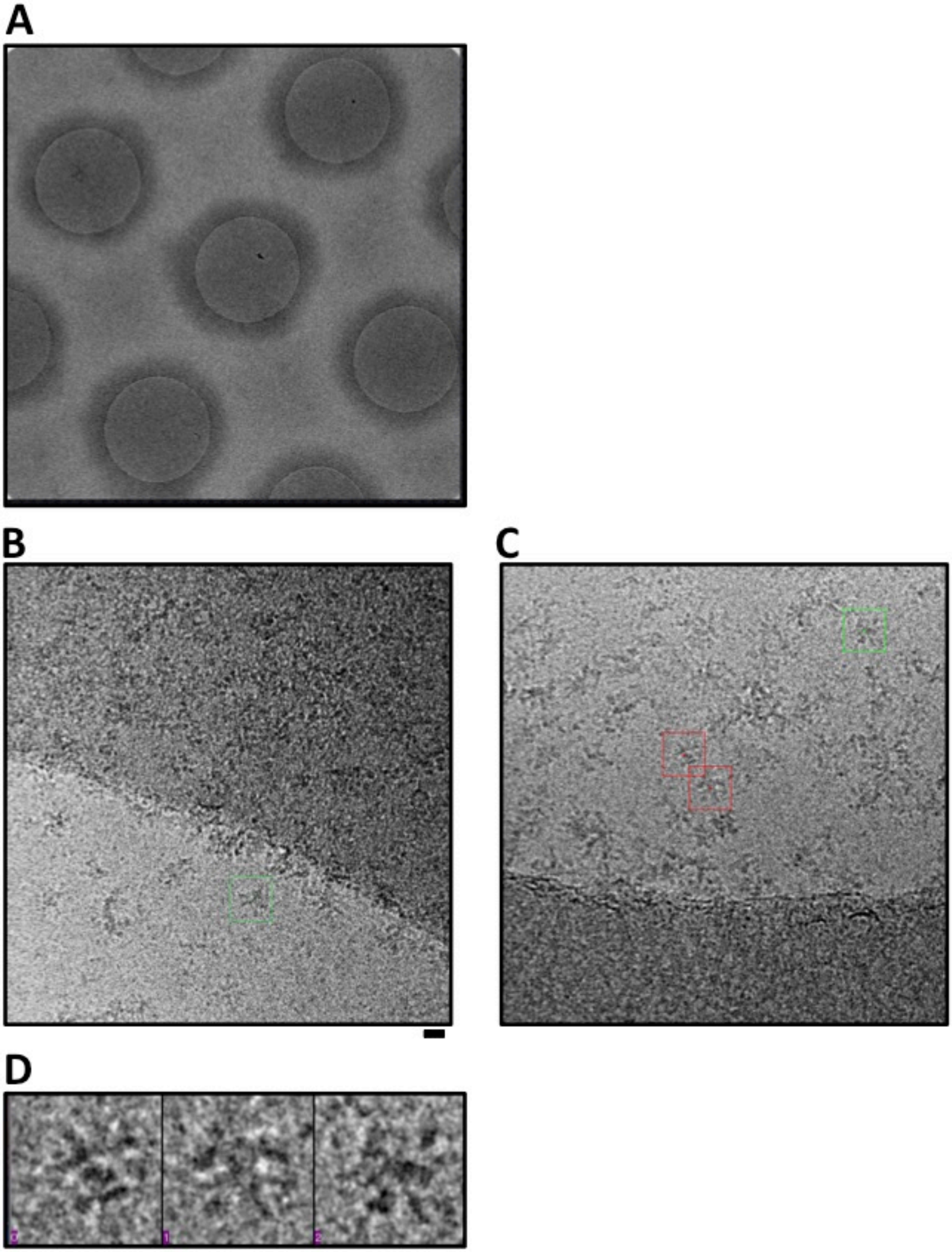

Figure S5

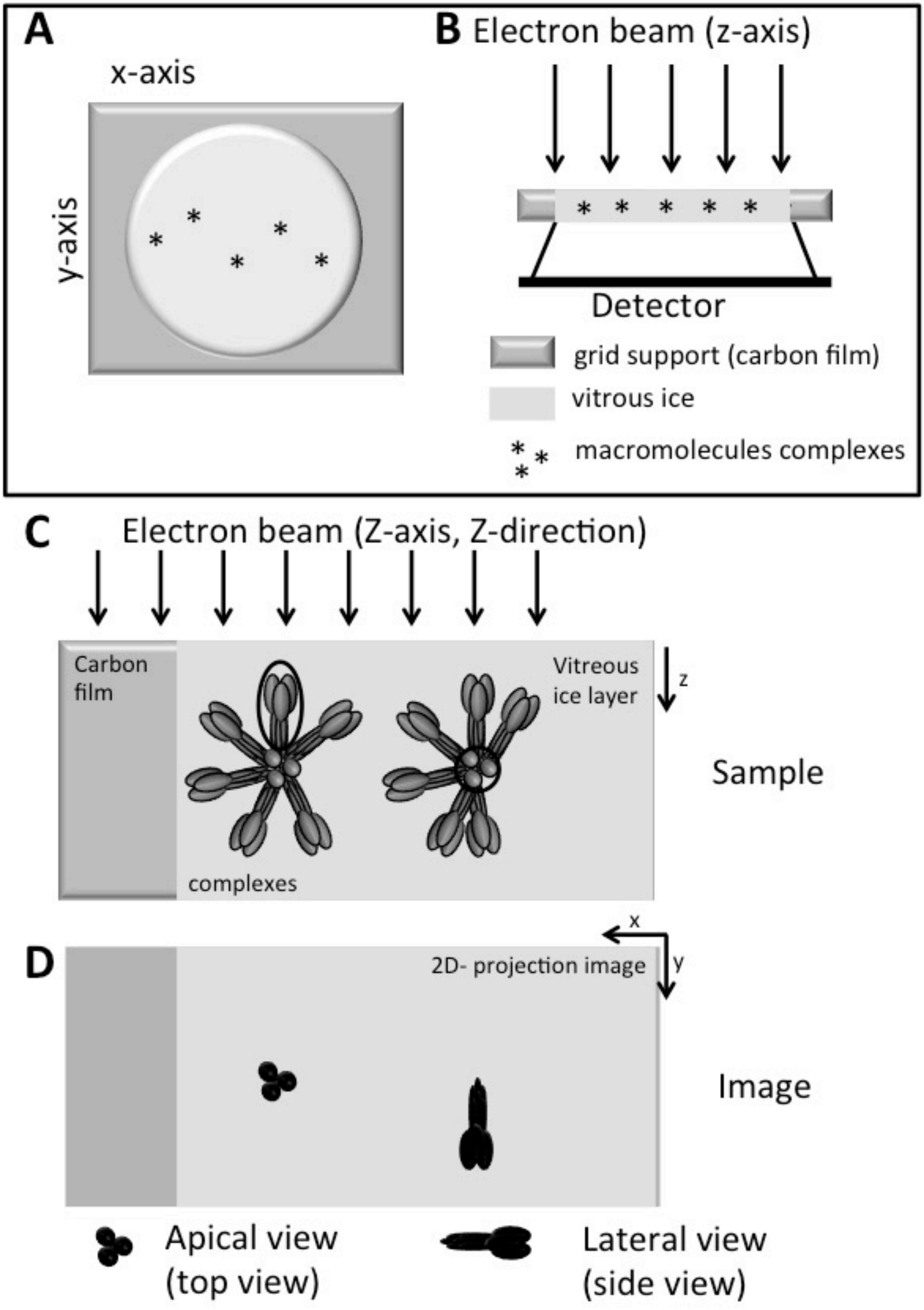
A
x-axis
y-axis
B Electron beam (z-axis)
Detector
grid support (carbon film)
vitrous ice
macromolecules complexes
C
Electron beam (Z-axis, Z-direction)
Carbon film
Vitreous ice layer
z
complexes
Sample
D
x
y
2D- projection image
Image
Apical view (top view)
Lateral view (side view)

Figure S6

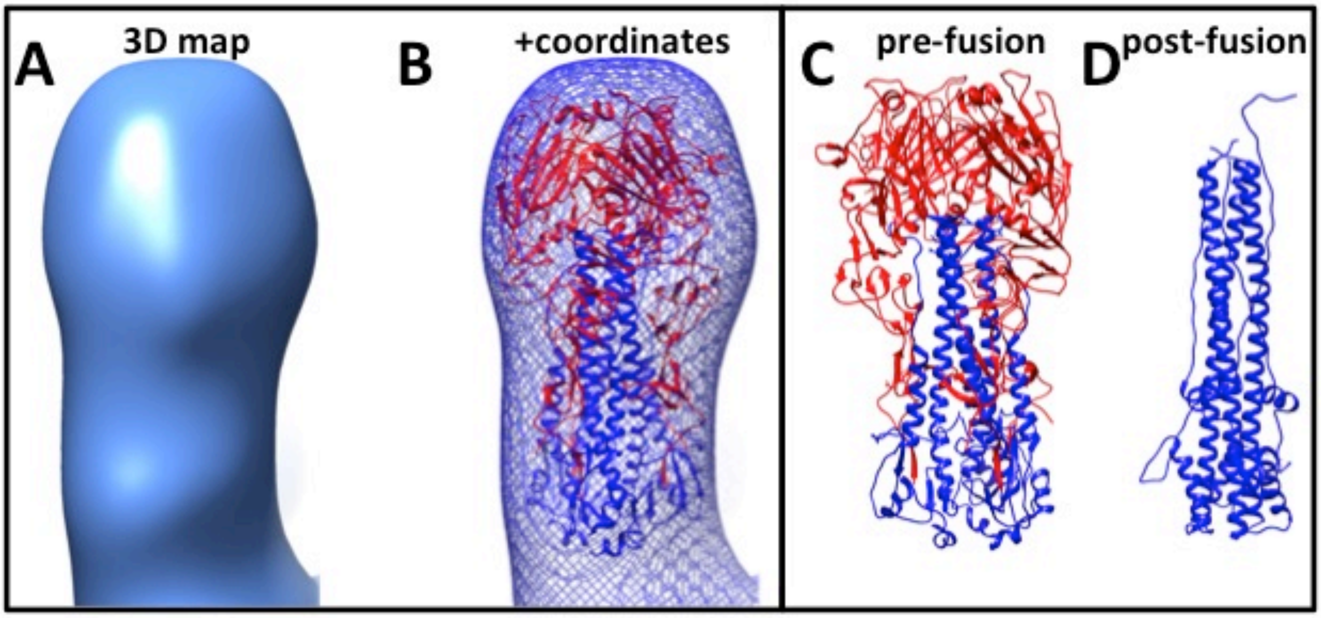

Figure S7

Supplemental Figure captions.

Figure S1. H7 sequence comparison. (A) Sequence alignment of H7 from A/Netherlands/219/2003 (H7N7) and A/Anhui/01/2013 (H7N9). Polybasic sites between HA1 and HA2 for A/Netherlands/219/2003 (H7N7) are highlighted in blue. The predicted transmembrane regions are shown in red. Cysteines are colored green.

Figure S2. Conformational dependence of mAb IN414 detection of H7 hemagglutinin probed by western blots. (A) Immunoblot of H7 proteins probed with mAb IN414. (B) Immunoblot of H7 proteins probed with ferret polyclonal sera to influenza H7N9 (IRR, FR-1250). Proteins were under reducing (+DTT) conditions during SDS-PAGE before transfer to nitrocellulose membrane. Proteins are H7 hemagglutinins from full-length A/Netherlands/219/2003 (H7N7) and A/Anhui/01/2013 (H7N9). Also, H7 ectodomain with His-tag and HA1 with His-tag for A/Netherlands/219/2003 were used. Molecular weight standards are in lanes 1 and 4.

Figure S3. Disulfide dependence of mAb IN414 detection of H7 hemagglutinin. (A) Immunoblot of H7 proteins probed with mAb IN414 under non-denaturing, reducing conditions (+DTT). (B) Immunoblot of H7 proteins probed with mAb IN414 under non-denaturing, non-reducing conditions. Proteins are H7 hemagglutinins from full-length A/Netherlands/219/2003 (H7N7) and A/Anhui/01/2013 (H7N9). Also, H7 ectodomain with His-tag and HA1 with His-tag for A/Netherlands/219/2003 were used. Molecular weight standards are in lanes 1 and 4.

Figure S4. Analysis of relative band number and distances of HA ladder by profile analysis of immunoblot. (A) Immunoblot of H7 proteins probed with mAb IN414 under non-heating and non reducing conditions. Proteins are H7 from A/Anhui/01/2013 (H7N9) and A/Netherlands/219/2003 (H7N7) and are denoted as H7(A) and H7(N), respectively. Lane 1

contains molecular weight standards. Yellow rectangles represent areas used to obtain 1D density trace profiles shown in panel B. (B) 1D profile traces of lanes in panel A. The sizes of the molecular weights standards are labeled. (C) Zoomed-in view of profiles of H7 proteins with estimated baselines. The relative peaks are labeled.

Figure S5. Examples of the distribution of H7 complexes by cryo-electron microscopy. (A) cryo image of a holey carbon grid with HA H7 complexes a low magnification (3800x) showing at low magnification that whether or not complexes are in a region of interest cannot be detected and requires higher magnification. Holes are circular regions. (B) Cryo image of HA H7 complexes with some complexes on the carbon film (dark grey) and in the vitreous ice (light grey), magnification (59000x). One of the complexes in the ice is boxed with a green square. Scale bar 20 nm. (C) cryo image of HA H7 complexes with lesser complexes on the carbon film (dark grey) and more in the vitreous ice (light grey) when compared to panel A. Some of the HA complexes in the ice are boxed with squares. (D) Examples of boxed HA complexes (particles) from panel B. Hemagglutinin H7 is A/Anhui/01/2013 (H7N9). Protein is represented as black (dark contrast).

Figure S6. Schematic of holey carbon film and particles in vitreous ice by cryo-electron microscopy. (A) Schematic of a region of holey carbon film viewing down the z-axis, which is parallel to the direction of the electron beam. Thus, the viewing direction of the viewer is that of the electron beam traveling through the sample down the z-axis. Axes x and y are indicated. (B) Schematic of a rotated view of the region of holey carbon film shown in panel A. The z-axis is parallel to the direction of the electron beam (denoted by arrows). Particles are not flat but are suspended in a layer of vitreous ice (frozen-hydrated state). The carbon film is shown as grey with the vitreous ice as light grey. The particles are represented as asterisks. (C) Enlarged schematic showing schematics of HA-complexes in a layer of vitreous ice. Select HA molecules that have a 3-

fold axis parallel to the electron beam (oval) and a 3-fold axis perpendicular to the electron beam (circle) are indicated within the schematics of the HA-complexes. (D) Schematic interpretation of HA orientation in 2D images based on projection features. The HA molecule with a 3-fold axis parallel to the electron beam would project an image with a three-dotted appearance that can be interpreted as an apical (top) view of a HA molecule. Likewise, the HA molecule with a 3-fold axis perpendicular to the electron beam would project a bi-lobed, peanut shaped image that can be interpreted as a lateral (side) view of the HA molecule. For clarity the projected 2D images are of only one HA molecule from each complex. The HA molecules at other angles relative to the electron beam would project images that would be size and shape combinations of dotted and peanut shaped patterns.

Figure S7. 3D reconstruction (3D map) of HA compared with pre-fusion and post-fusion HA ectodomain coordinate structures. (A) The HA 3D map is shown as a solid surface rendering. (B) The 3D map is shown as a wire mesh with docked coordinates of the pre-fusion structure of H7 ectodomain (PDBID 4N5J). HA1 is in red while HA2 is in blue ribbons, respectively. The transmembrane domain is not shown because no coordinates for them are available. (C) Examples of HA ectodomain coordinates in a pre-fusion state (PDBID 4N5J) and in a (D) post-fusion state (PDBID 1QU1). The post-fusion state lacks globular HA1 regions found in the pre-fusion state. The pre-fusion state is larger in width and has globular HA1 regions at the top when compared to the post-fusion state (C vs. D). Based on size and shape the pre-fusion state coordinates match the molecular shape of the HA 3D map from cryo-electron microscopy (Panels A and B).
